# Quality criteria of involuntary psychiatric admissions - before and after the revision of the civil code in Switzerland

**DOI:** 10.1186/s12888-016-0998-z

**Published:** 2016-08-12

**Authors:** Isabelle Kieber-Ospelt, Anastasia Theodoridou, Paul Hoff, Wolfram Kawohl, Erich Seifritz, Matthias Jaeger

**Affiliations:** 1 University of Zurich, Zurich, Switzerland; 2 Department for Psychiatry, Psychotherapy and Psychosomatics, University Hospital of Psychiatry, Lenggstrasse 31, P.O. Box 1931, 8032 Zurich, Switzerland

**Keywords:** Involuntary admission, Mental disorder, Psychiatric hospital, Quality

## Abstract

**Background:**

The goal was to investigate the quality in terms of formal and content-based comprehensiveness of the forms for involuntary admission before and after the introduction of the new law (KESR, “Kindes- und Erwachsenenschutzrecht”) for the regulation of involuntary admission. Moreover, the study aimed at assessing if the quality of the admission forms was associated with the professional qualifications of the professionals ordering them. Finally, the patients were characterized.

**Methods:**

Retrospective evaluation of all commitment reports at the University Hospital of Psychiatry within a six month period before and after the introduction the KESR (N(2012) = 489; N(2013) = 651). Formal and content-related criteria for the commitment certificates were recorded as well as the socio-demographic and clinical data of the cases admitted. There were no exclusion criteria. The data was descriptively evaluated, formal and content-based criteria were compared between groups of admitting professionals. The Chi-Square-Test following Pearson and *T*-Test were used to test for group differences.

**Results:**

Formal and content-related quality criteria deficiencies were noted. The best-documented forms came from psychiatrists and emergency physicians, followed by general practitioners and hospital doctors. There have been improvements in the quality of the documents since the new KESR within all professional subsamples.

**Conclusions:**

Psychiatrists and those who regularly deal with emergency commitments were likely to issue forms of high quality. Due to the considerable consequences associated with involuntary admission for affected individuals, their relatives and also professionals, the considerable deficits in the quality of the documentation must be intensively addressed in training, advanced training, continuing education and in daily routines.

## Background

Admission without the consent and/or against the will of an individual to a psychiatric institution for in-patient treatment, supervision, observation or assessment, always constitutes a significant interference with individual rights and is therefore subject to strict legal regulations [1]. Until 2012, in Switzerland, the formal and content-based prerequisites for the involuntary admission of a person to an in-patient medical facility were regulated in Art. 397 of the Swiss Civil Code (ZGB). In general, the local public authority was responsible, but could delegate the responsibility to admit to medical doctors [2]. This practice resulted in a high variability in Cantonal involuntary admission-ratios (e.g. in 2009: Aargau 37 %, Zurich 26 %, Geneva 4 % [3, 4]). Furthermore, there was significant variability in formal and content-based quality of the admissions between different professional groups of admitting physicians. A study from 2001 confirmed that only 21 % of the involuntary admission reports evaluated in an in-patient hospital in Canton of Zurich complied with all formal and content-based requirements. The quality of the reports issued by psychiatrists was by far the highest, followed by non-psychiatric hospital doctors and general practitioners (GP) [5]. A study from 2014 illustrates that there are still considerable deficiencies with respect to formal and content-based quality criteria in the involuntary admission reports and variations in socio-demographical and clinical characteristics of the patients committed by different groups of physicians [6].

In 2013, a revised federal legislation (abbreviated KESR) was introduced that regulates the prerequisites for involuntary commitment as well as further details that are associated with involuntary hospitalization and coercive practices [7]. According to Art. 426 ZGB, a person who suffers from mental illness, mental retardation or severe neglect and evinces a need for protection (“if the necessary treatment or care cannot be provided otherwise”) can be admitted to a “suitable” facility such as a psychiatric hospital [8]. The conflict between the right to treatment, preservation of personal rights to liberty and protection of society as a whole, demands the most precise rules in order to assure a legitimate practice [6]. The new KESR attempts to deal with current international health policy concerns, namely the reduction of involuntary commitment and treatment against the patient’s will as well as strengthening human rights in psychiatric treatment, e.g. by introducing the possibility for all patients to issue advanced directives and the involvement of a personal representative for individuals who were involuntarily admitted [9, 10].

However, the Cantons still have the opportunity to delegate the responsibility to admit patients involuntarily to medical doctors, and most Cantons made use of that possibility. The local public authorities are only obliged to review the legitimacy of involuntarily admissions by physicians after a duration of six weeks [8]. Therefore, it is likely that the variability in formal and content-based quality of the admissions between different professional groups of admitting physicians would persist.

With respect to the high rates of involuntary admissions in Switzerland this study aims at reviewing if the documentations of the admissions are according to the prerequisites as demanded by the law. The study therefore pursues the quality of involuntary admissions into a psychiatric clinic before and after the revision of the civil code based on formal and content-related criteria as documented in the admission forms. As opposed to the old legislation before 2013, the new KESR specifies more detailed, what criteria have to be examined before a person can be involuntarily admitted. Also, the law emphasizes that these obligatory criteria have to be documented in the admission form. Therefore, the admission forms were chosen as a suitable indicator for the quality of the admission procedure. As opposed to the basic old forms (that only included sociodemographic data of the patient admitted as well as space for the justification of the admission), the new forms include a more elaborated structure as well as an instruction concerning the obligatory criteria that have to be included in the justification for the admission. The data shown here, expand on formerly published results on quality of admissions before the revision of the law [6] with a second wave of data collection after the introduction of the new legislation KESR. Due to specification of the mandatory criteria for involuntary admission in the KESR and the obligation to document these criteria on the admission forms it was hypothesized that the quality of the commitment forms after the introduction of the new law would be higher than before, and that a classification corresponding to the professional qualification of the assigning person still could be observed [6]. In addition, the patients involuntarily admitted are characterized with respect to socio-demographic and clinical parameters. According to the literature, male gender, a low level of education, unemployment, and diagnosis of psychosis and dementia in old age are viewed as risk factors for involuntary admission to a psychiatric hospital [11–13].

## Methods

### Procedures

This study is based on a retrospective evaluation the quality of admission forms in terms of formal and content-based comprehensiveness as well as the medical files of all patients admitted to the Zurich University Hospital of Psychiatry (PUK) in the collection period of six months before (March to August 2012) and after (June to November 2013) the revision to the civil code (KESR). With a greater metropolitan catchment area of 485,000 residents the Clinic for Psychiatry, Psychotherapy and Psychosomatics at PUK (KPPP) provides 345 beds and constitutes the largest clinic for adult psychiatry in Switzerland. In 2013 around 4500 patients were admitted (3600 in 2012) to KPPP, around a third of them by involuntary admission [14, 15]. The commitment documents as well as parts of the medical records were evaluated by the first author of this manuscript in 2014 on the basis of a standardized criteria catalogue. The socio-demographic and clinical data concerning the course of treatment after admittance to the hospital, some details of the first two days of the stay as well as the conditions on release were collected from the patient files. All patients’ data were managed and assessed anonymously. The study was reviewed and approved by the Cantonal Ethics Commission (Ref.-No. EK: 2012-0149; Decision from 09.03.2012).

### Criteria catalogue

The admissions documents were evaluated according to the following criteria using open ended responses and multiple choice items where suitable:

- **Category of issuing party**: Psychiatrist (medical specialists in private practice, emergency psychiatrist), psychiatric institution (hospital, out-patient clinic, consulting service), non-psychiatric hospital physician, GP (doctor in general medicine, non-psychiatric medical specialist in private practice), emergency physician (“SOS-Ärzte”, a private, out-patient emergency medical company that carries out a significant portion of involuntary admissions in Canton of Zurich). Individuals admitted directly by authorities (i.e. Kindes- und Erwachsenenschutzbehörde, KESB) were excluded from the evaluation due to the very small number of cases.
- **Formal criteria**: The patient’s personal data (name, date of birth, residence), personal medical examination (place, date), personal data of the committing party (name, address, function), instruction on legal recourse, signature.
- **Content**-**based criteria**: Type of involvement in the case (e.g. as emergency psychiatrist, during consultation in office practice), medical history information, current situation, mental state, environmental factors (taking into account family members and the social network), justification for in-patient treatment, purpose for in-patient admission (e.g. treatment, clarification, monitoring) and notification of a person of trust.
- **Justification for involuntary commitment**: Danger to self, danger to others, severe self-neglect, considerable burden to the environment.
- **Cause for involuntary commitment**: Disorientation/delirium/dementia, intoxication/substance abuse, psychosis/mania, depression, suicidal ideation/self-injury, aggression, refusing food/necessary somatic treatment or other behaviour hazardous to health. These categories are based on syndromal/behavioral descriptions in the admission forms, not on nosological categorization according to ICD-10.

In addition, the following socio-demographic and clinical data as well as characteristics of the progression of the stay in the hospital are obtained from the patient’s medical file: Age, gender, main and supplemental diagnoses (according to ICD-10), number of earlier hospitalizations in the PUK, time of admission (day-time, night-time), duration of stay, coercive measures within the first 72 h after admission (isolation/restraint, forced medication, intensive monitoring/permanent supervision, quarter-hourly to hourly contacts with nurses), judicial hearings and conditions on release (regular release in mutual agreement or extraordinary release e.g. against medical advice, escape, release on court decision). Chart review was only used to describe the sample, not to evaluate the appropriateness of the admission.

### Assessment

In each case, the stamp of the practice gave information about the party ordering commitment. To be able to assess the qualitative comprehensiveness of the admissions, five formal and eight content-based quality criteria for the admission documents were evaluated by the first author using a three-level scale (0 = no information, 1 = incomplete information, 2 = complete information). First, the percentile shares of documents with complete information with reference to the categories of issuing parties were compared. In addition, sum values of the five formal criteria (maximum of ten points possible) as well as the seven content-based criteria (maximum of 14 points possible, only seven criteria were included in the sum value because “person of trust” was not a criterion before 2013) and the total quality were generated for each admission document.

### Statistical data analysis

The statistical assessment of the data collected was completed using the IBM SPSS Statistics Program, Version 21 (SPSS Inc., Chicago, USA). Along with the descriptive analyses of the total sample, group differences between the categories of professionals ordering commitment were analyzed. Categorical variables were compared between subsamples using cross-tables and Chi-Square-Tests following Pearson, continuous variables were compared using mean comparisons with *T*-Test. Results with a *p*-value of less than 0.05 were considered significant.

## Results

The results section mainly reports data from the 2013 sample (after revision of the civil code). However, it refers to some data from the 2012 sample since it was hypothesized that the quality of the admission forms improved after the introduction of the new law. The most relevant 2012 data is therefore included in the following section. The detailed results of the 2012 sample were published elsewhere [6].

### Samples

All cases of involuntary admission to PUK during both examined periods of six months were recorded in the clinic’s internal database (702 involuntary admissions in 2012 and 733 in 2013). Concerning the 2012 period, a total of 489 forms was included in the study, and the selection procedure was already described elsewhere [6]. The reason for the large discrepancy between the total number of involuntary admissions in the 2012 period and the admission included in the study was an administrative one. In 2012, all admissions were recorded by the administration (including geriatric psychiatry and rehabilitation patients treated on a different location of PUK). These admissions had to be excluded afterwards, while in 2013, only the selected target admissions could be provided by the administration.

In the 2013 period the files of 47 involuntary admissions (6.4 %) could not be found for assessment; 18 cases (2.5 %) were voluntary commitments erroneously recorded as involuntary. In four cases (0.5 %) the involuntary admission-form was not included in the file. 11 official documents were ordered by KESB; in two other forms the issuing party could not be assigned to any category. Thus, 82 cases were excluded from the assessment.

Finally, 1140 forms (489 before the revision of the civil code and 651 afterwards) issued by physicians from five categories were assessed (Table 1). In 2013, 54 patients were admitted more than once during the whole period (thereof 7 three and 2 four times).

**Table 1:** Assigning professionals (2012 and 2013 samples; *N* = 1140)

	2012		2013	
	*N*	%	*N*	%
Psychiatrists	122	24.9	203	31.2
Psychiatric institutions	81	16.6	59	9.1
Hospital doctors	124	25.4	172	26.4
General practitioners	64	13.1	92	14.1
Emergency physicians	98	20.0	125	19.2
Total	489		651	

### Formal criteria

Formal criteria of the admission forms of the 2013 sample are displayed by professional group of the admitting physician in Table 2. The patient’s personal data were documented in 96 % of cases and the signature in 99 %. The personal medical examination (place and date) was better documented in 2013 compared to 2012 (95 % vs. 86 %; *p* < 0.001) [6]. Concerning this criterion, the hospital doctors, emergency physicians and the GPs achieved considerably better values than in pre-revision documents. Personal data of the issuing party were significantly better recorded in 2013 (94 % vs. 91 %; *p* = 0.022) [6]. Better results were also achieved in the documentation of the instruction on legal remedy issued (79 % vs. 33 %; *p* < 0.001) [6]. Total sum values for the five formal criteria for involuntary admission documents was 9.4 out of 10 points, without significant differences between the categories of assigning parties (see also Table 4).

**Table 2:** Formal criteria for involuntary admission documents *(2013 sample; N = 651)*

	Psychiatrists	Psychiatric Institutions	Hospital doctors	General practitioners	Emergency physicians	Chi^2^
Patients’ personal data	97.0 %	91.5 %	95.3 %	95.7 %	95.2 %	12.00
Patient examination	94.6 %	94.9 %	91.9 %	95.7 %	98.4 %	7.95
Personal data of issuing party	94.1 %	84.7 %	94.2 %	96.7 %	96.8 %	14.43
Instruction on legal remedy	74.4 %	74.6 %	85.5 %	70.7 %	84.8 %	13.92*
Signature	100.0 %	96.6 %	99.4 %	98.9 %	99.2 %	7.10

Chi-Square-Test: *p < 0.05

### Content-based criteria

Content-based criteria of the admission forms of the 2013 sample are displayed by professional group of the admitting physician in Table 3. The “current situation”, “psychiatric findings”, “justification of in-patient admission”, as well as the “purpose of the involuntary commitment” were well documented in most documents. The group of GPs evinced deficient results in 15 %, and hospital doctors in 10 % concerning documentation of the mental state and psychopathology. Information about medical history was recorded on average in 70 % of the forms. The hospital doctors issued fewer than half of the involuntary admission-documents with respect to the medical history. The contact data for trusted third parties was documented in nearly a third of all involuntary admission-documents issued after the revision.

**Table 3:** Content-based criteria of involuntary admission documents *(2013 sample; N = 651)*

	Psychiatrists	Psychiatric Institutions	Hospital doctors	General practitioners	Emergency physicians	Chi^2^
Involvement	36.0 %	67.8 %	33.7 %	17.4 %	51.2 %	95.93**
Medical history	79.3 %	88.1 %	48.8 %	62.0 %	75.2 %	58.38**
Current situation	100 %	100 %	99.4 %	100 %	100 %	2.79
Mental state	94.1 %	94.9 %	90.1 %	84.8 %	93.6 %	20.33*
Social network	44.8 %	44.1 %	16.9 %	23.9 %	35.2 %	56.69**
Justification for IC	97.0 %	96.6 %	95.3 %	94.6 %	96.8 %	4.06
Purpose of IC	94.6 %	91.5 %	87.8 %	89.1 %	94.4 %	9.68
Person of trust	39.9 %	32.2 %	29.7 %	32.6 %	20.0 %	26.38*

Chi-Square-Test: *p < 0.05 **p < 0.001

### Comparison of formal and content-based criteria sum scores before and after the revision of the legislation

Table 4 depicts the sum scores of formal and content-based criteria as well as a total score before and after the revision of the legislation (i.e. the 2012 and 2013 samples). The formal quality of the 2012 form met the criteria at overall rates of 80–90 % depending on the group ordering commitment. Concerning content-based quality results of between 57 and 74 % were achieved. The total values of the content-based criteria were highest in the group of psychiatric institutions and lowest for hospital doctors (Table 4) while hospital doctors and GPs scored 1.5 points below the overall average.

**Table 4:** Total values of formal and content-based criteria (2012 and 2013 samples; *N* = 1140)

	Psychiatrists	Psychiatric Institutions	Hospital doctors	General practitioners	Emergency physicians
Formal criteria 2012	8.98	8.27	8.48	8.06	8.44
Formal criteria 2013	9.32	9.08	9.47	9.24	9.58
T	−2.78*	−3.53**	−6.76**	−6.34**	−9.55**
df	323	138	294	154	221
Content criteria 2012	9.68	10.32	8.08	8.69	9.54
Content criteria 2013	11.22	11.85	9.80	10.10	11.46
T	−6.54**	−3.76**	−6.44**	−3.56**	−8.15**
df	323	138	294	154	221
Total 2012	18.66	18.59	16.56	16.75	17.98
Total 2013	20.54	20.93	19.27	19.34	21.05
T	−6.54**	−4.45**	−8.95**	−5.46**	−10.65**
df	323	138	294	154	221

Range of scores: Formal criteria 0–10; content-based criteria 0–14, total 0–24

*df* degrees of freedom

*T*-Test: *p < 0.05 **p < 0.001

In 2013, the complete formal quality was met in 91–96 % of the forms while the content-based criteria were met in 70–85 %. Since the possibility of involving a person of trust was only introduced with the new legislation, the comparison of the content-based criterion “notification of a person of trust” before and after the revision is not possible. Consequently, only seven content-based criteria were compared. The total values of the content-based criteria were likewise best fulfilled by the group of psychiatric institutions with an average of 12 out of a maximum of 14 possible points. The hospital and emergency doctors achieved the lowest values with an average of 10 points. The best total sum values were achieved by the emergency physicians with 21 out of a maximum of 24 points, followed closely by the group of psychiatric institutions and psychiatrists. GP and hospital doctors produced the least results with 19 points (Table 4). In all subgroups, however, significantly better results could be obtained in 2013 than in 2012 (*p* < 0.001).

### Justifications for involuntary admissions and characteristics of committed patients

The distribution of purpose for involuntary admission, causes of threat as well as socio-demographic and clinical characteristics are shown in Table 5. In the 2013 sample, admissions were significantly more frequently mandated due to severe self-neglect than 2012 (16 % vs. 10 %; *p* = 0.007). Concerning causes of threat, there were no significant changes. The decline in “suicidal ideation/self-injury” and the increase in “refusal of food/necessary treatment” can be regarded as a statistical trend only.

**Table 5:** Socio-demographic and clinical characteristics of participants, comparison of samples

Variable	2012 sample (*n* = 489)		2013 sample (*n* = 651)		Chi^2^- Test T-Test^a^		
	*n*	%	*n*	%	Chi^2^	df	*p*
	Mean^a^	SD^a^	Mean^a^	SD^a^	T^a^		
Age^a^	45.58	20.12	48.1	19.40	4.548	1	0.033
Male	269	55.0	333	51.2	1.668	1	0.197
Previous hospitalizations^a^	3.67	6.14	4.93	7.72		1	0.003
Day-time admission	258	52.8	361	55.6	0.922	1	0.337
Duration of stay (days)^a^	21.41	23.84	26.16	27.28		1	0.002
Reason for compulsory admission					10.49	5	0.063
Self-endangering	407	83.2	542	83.3	0.000	1	0.991
Endangerment of others	203	41.5	243	37.3	2.055	1	0.152
Severe self-neglect	49	10.0	101	15.5	7.377	1	0.007
Stress for their families and friends	38	7.8	53	8.1	0.052	1	0.819
Clarification, assessment	9	1.8	27	4.1	4.860	1	0.027
Cause of threat							
Disorientation, delirium, dementia	92	18.8	105	16.1	1.408	1	0.235
Intoxication, substance abuse	159	32.5	215	33.0	0.033	1	0.856
Psychosis, mania	219	44.8	302	46.4	0.290	1	0.590
Depression	73	14.9	96	14.7	0.007	1	0.932
Suicidal tendency, self-injury	195	39.9	227	34.9	3.004	1	0.083
Aggression	206	42.1	270	41.5	0.049	1	0.825
Refusal of food intake, medical treatment	97	19.8	158	24.3	3.162	1	0.075
Primary diagnosis					16.906	9	0.050
organic disorder (F0)	63	13.0	85	13.1			
substance-related disorder (F1)	109	22.5	123	18.9			
schizophrenic disorder (F2)	147	30.3	232	35.6			
affective disorder (F3)	77	15.9	110	16.9			
neurotic disorder (F4)	55	11.3	67	10.3			
Seclusion/restraint	84	17.2	124	19.0	0.654	1	0.419
Forced medication	59	12.1	89	13.7	0.637	1	0.425
Monitoring					17.154	4	0.002
Every hour	10	2.0	33	5.1			
Every half an hour	26	5.3	36	5.5			
Every quarter of an hour	22	4.5	33	5.1			
Permanent supervision	18	3.7	51	7.8			
Appeal	42	8.6	53	8.2	0.069	1	0.793
Discharge from hospital					1.758	4	0.780
regular discharge	446	91.2	587	90.2			
against doctor’s orders	11	2.2	23	3.5			
Escaped	23	4.7	31	4.8			
release by court decision	6	1.2	7	1.1			
Others	3	0.6	3	0.5			

*df* degrees of freedom

^a^Mean, SD (standard deviation), *T*-Test where indicated

Fifty-three percent of all cases of involuntary admissions were male. The average age was 45.6 years in 2012 and 48.1 years in 2013 (*p* = 0.033). Schizophrenia and related disorders (F2) was the most frequent main diagnosis according to ICD-10, followed by substance-related disorders (F1), and affective disorders (F3). The proportion of schizophrenic disorders increased from 30 % in 2012 to a solid 36 % in 2013 (*p* < 0.05). The substance-related disorders decreased from 23 to 19 %.

In the 2012 sample, the number of earlier hospitalizations was 3.7, and significantly higher in 2013, i.e. 4.9 (*p* = 0.003). The average duration of hospitalization was 21.5 days in 2012 and 26 days in 2013 (*p* = 0.002). Involuntary admission during the daytime (between 8 am and 8 pm) in 2012 accounted for 52.8 % and 55.6 % in 2013. In 2013, the involuntarily admitted had to be placed under observation more frequently (*p* = 0.002). Particularly, permanent supervision was documented more frequently in 2013 (7.8 % vs. 3.7 % in 2012). Also, hourly supervision was more frequent used in 2013, rising from 2 to 5.1 %. It is worth noting that discharges against medical advice increased slightly but not significantly, from 2.2 to 3.5 %.

### Introduction of obligatory review of FU after six weeks by local public authority

As we found in the present study that in general the duration of stay among involuntarily admitted subjects increased from 21 to 27 days on average in contrast to the intention of the law, we additionally examined the percentage of cases that stayed longer than six weeks. Regarding the high percentage of individuals with primary diagnoses of schizophrenic disorders among those patients (increased after the revision from 30 to 36 %), we differentiated between subjects with schizophrenic disorders and other primary diagnoses (Table 6). The percentage of individuals with an involuntary hospitalization longer than 6 weeks was much higher among individuals with schizophrenia main diagnosis (increase from 21 to 26 %) compared to other diagnoses (that remained stable at 15 %).

**Table 6:** Involuntary admissions longer than six weeks, in subjects with schizophrenia and related diagnoses compared to subjects with other primary diagnoses, before and after the revision

	2012 sample (*n* = 489)					2013 sample (*n* = 651)				
Variable	Hospitalization longer than 6 weeks		Chi^2^	df	*p*	Hospitalization longer than 6 weeks		Chi^2^	df	*p*
	No	Yes				No	Yes			
	n (%)	n (%)				n (%)	n (%)			
F2 primary diagnosis										
No	284 (85.5 %)	48 (14.5 %)	3.437	1	0.064	3338 (85.1 %)	59 (14.9 %)	13.162	1	<0.001
Yes	123 (78.8 %)	33 (21.2 %)				187 (73.6 %)	67 (26.4 %)			

## Discussion

This study confirms that in clinical practice the procedure of involuntary admissions before and after revision of the civil code was primarily carried out by physicians in the Canton of Zurich. Less than 2 % of all involuntary admissions were issued by the responsible public authorities. According to Articles 428 and 429 of current ZGB, the authorities must review the involuntary admission mandated by physicians after six weeks in order to meet the affected party’s right to a uniform legal proceeding [8]. It is worth noting that nearly every fifth involuntary admission stayed longer than six weeks after the revision and even more if there was schizophrenic disorder as primary diagnosis, i.e. 26 % (Table 6). This suggests that the new law did not have the intended effect of reducing the duration of stay, and potentially an adverse effect on individuals with schizophrenia. However, we cannot state causality based on the present data and do not know, how many patients actually stayed longer than six weeks under FU conditions. This is due to the documentation of the admission status only and not the course of voluntariness, i.e. if and when a patient signed to remain in the hospital under voluntary conditions.

According to Art. 430 ZGB of the new law, the committed person must be examined personally by a doctor with a note made of the place and date. In addition, the decision to commit must at least include the name of the committing person, the mental state, the purpose of the commitment and also an instruction concerning legal recourse. The Department of Health of the Canton of Zurich and other stakeholders consequently issued a new form for the purpose of ordering FU [8, 16]. The formal requirements and the explanations on the new pre-printed forms for involuntary admission in use after the revision to the civil code also might facilitate a simpler, and qualitatively better documentation. In summary, both the new form and the new legislation improved the quality of the involuntary admissions.

### Qualitative criteria of commitment forms

In the Canton of Zurich, under a cantonal implementation law, every doctor licensed to practice can independently order an involuntary admission, in spite of their widely diverging professional experiences and qualifications. In the draft law for the revision to the civil code and also in the prior requirements of the Swiss Academy of Medical Sciences (SAMW) the question was discussed, whether this authority should be restricted exclusively to experienced and/or specially trained doctors, e.g. psychiatrists [1, 17]. However, this suggestion was not taken up by parliament [17], although another study came to the conclusion that the rates of involuntary admissions were lower if only specialized doctors were allowed to order involuntary commitment or when an independent service for legal assistance was established [3].

The results of the 2012 sample have already been presented and discussed in detail elsewhere [6]. There were considerable deficiencies in formal and especially content-based quality criteria as well as marked differences between the different subgroups of physicians ordering commitment [6]. Most strikingly, only a third of the documents indicated whether the instruction on legal remedy was given or not. This can be regarded as a major deficiency since the Constitution of the Swiss Confederation (BV) itself states the right to explanation of the purpose for involuntary detention as well as legal remedies (Art. 31 BV [18]). In the 2013 sample, significantly better results could be achieved concerning instruction on legal remedy with around 80 % (*p* < 0.001). However, this is most likely due to the introduction of a check box for this purpose on the new form [16].

The four other studied formal criteria (“patient’s personal data”, “personal medical examination”, “personal data of party ordering commitment” and “signature”) were addressed in over 86 % of the forms in 2012 and more than 94 % of the forms in 2013. Nevertheless, the deficient documentation in 6 % of forms must be viewed critically since involuntary admissions constitute a considerable restriction on the patient’s autonomy, especially on his/her freedom of movement and the protection of his/her privacy (Art. 10 and 13 BV [18]) and are associated with comparatively low administrative expense. This is even more important since compliance with the formal requirements serves to protect the affected party from unjustified or premature commitment [19]. Corresponding criteria are listed in the KESR (Art. 426), in the Cantonal Patient Protection Act and in the Medical Ethics Guidelines of the SAMW [1, 8, 20].

In 2012, inadequate documentation was found in more than 50 % of the content-based criteria. The criteria “current situation”, “psychiatric finding” and “justification for involuntary admission before the revision” were documented in more than 95 % of the documents. However, the other content-based criteria were not completed in a large portion of the commitments [6]. In the 2013 sample, the criteria “involvement” and “purpose of the FU” improved significantly. Reporting the purpose of commitment is easier on the new forms because of a better structure and more instructions, and the person ordering commitment can just set a mark at “treatment” or “supervision”.

The commitment forms showed considerable differences between the professional groups of physicians ordering commitment with respect to the formal and content-based quality criteria. In 2012, the psychiatrists and psychiatric institutions achieved the highest quality, followed by the emergency physicians, while the GP and hospital doctors evinced the largest proportion of deficient commitment documents [6]. All subgroups achieved better results in 2013. The emergency physicians achieved slightly better results than the psychiatric institutions as well as psychiatrists. The GP and hospital doctors again evinced the least completely documented forms.

These results indicate that specialized doctors in psychiatry are more experienced and practiced in this area than their non-psychiatric colleagues [5]. Non-psychiatrists scarcely perform involuntary commitment, and this did not change with the revision of the civil code as no qualitative standard for those executing FU has been implemented. One indication of the relevance of daily routine is the high quality of documentation in the forms by emergency physicians. The latter is indicative of a routine handling of the instrument of involuntary admissions, since a high proportion of emergency physicians do not have any training as specialists in psychiatry. According to sections Art. 27 and 30 of the Implementation Act for the new law of the Canton of Zurich (EG KESR), the authority of the ordering doctors, however, is not granted by a qualification in a medical specialization or demonstration of a regular practice; instead the practicing doctors must only be subject to “regular continuing education” in this field [21]. In the training of all doctor groups, it should be possible to increase the significance of the topic, a more nuanced content-based reflection on the particularities of the diverse commitment situations and with it the quality of daily practice.

### Socio-demographic and clinical characteristics

The distribution of gender in cases of involuntary admission was 53 % male and 47 % female. With respect to the percentile distribution of the primary diagnoses according to ICD-10, schizophrenic disorder came up most frequently, i.e. more than 30 %. This diagnosis is, according to Link et al. [22] and Steinert [23], often associated with aggressive and violent behavior. Based on the professional literature, it was expected that the two risk factors, male gender and diagnosis of a psychosis, would accompany involuntary clinic admission [11–13, 24].

According to the new law, the local public authority is obliged to review after six weeks if the involuntarily hospitalized patients admitted by physicians must stay in the clinic on the basis of FU. This regulation was introduced in order to foster shorter durations of stay under involuntary conditions. We therefore examined the percentage of cases that stayed longer than six weeks. The continued assessments showed that in the case of a diagnosis of schizophrenic disorders every fourth commitment had to be extended or was longer than six weeks on a voluntary basis (this could not be distinguished based on the available data). However, we do not know if all those individuals were subject to the revision procedure by the local public authority or the share of those who changed their status to voluntary hospitalization.

### Limitations

The high amount of missing data in 2012 up to one quarter of the suitable documents must certainly be considered a limitation, especially since there was limited access to patient files at the time the study was conducted [6]. Files for disability-fund-clarification were often tied up for a period of several months and certain patient cases that are registered in the internal database were not treated at the KPPP, but instead at other clinics or locations of PUK. These aspects could have been taken into consideration in the 2013 sampling, resulting in considerably less missing cases. Certainly it should be noted that the assessment of the criteria is subject to the influence of the investigator even if the data collection was based on a structured checklist. In addition, it must be noted that the data collected refers to the situation in the Canton of Zurich and cannot be amplified offhand to other Cantons or even countries due to different domestic laws and procedures concerning involuntary commitment practice.

## Conclusions

After the introduction of the KESR the documentation of involuntary commitment has improved. This might be due to the more precise formulations of formal and especially content-based criteria (especially Art. 430 ZGB) [3, 8] as well as the new, better structured form and continuing education for the doctors ordering commitment as mandated in the implementation act for the new law and carried out by PUK Zurich [16, 21].

The results of this study still point on the persisting need for improvement especially concerning content-based quality criteria. The revision of the legal basis did not result in any restriction of the authority for the decision on an involuntary admission in the Canton of Zurich resulting in a diverse clinical practice and considerable differences in the quality of FU between different groups of physicians. This study points to a higher quality of the documents issued by psychiatrists and physicians with more routine concerning the procedure. As long as the specific professional qualification does not become a primary criterion in the future for doctors ordering commitment, all groups of doctors, especially non-psychiatric specialists should devote special attention to this topic and specifically make sure to attend advanced and continuing education.

## Abbreviations

ANOVA, Analyses of variance; Art., article; BV, Constitution of the Swiss Confederation; e.g., for example; EG KESR, Implementation Act to the KESR in the Canton of Zurich; ff., and following; FFE, Involuntary admission before the revision of the KESR (until December 31, 2012) based on Art. 397 ff. ZGB (involuntary psychiatric commitment) with the “Guardianship Authority” as the formerly responsible authority; FU, Involuntary admission after the revision of the KESR (since January 1^st^, 2013); GP, general practitioners; i.e., this means; KESB, The local public authority for the Protection of Minors and Adults [hereafter also referred to as the “responsible agency”] after the Revision of the Civil Law; KESR, Law for Protection of Minors and Adults (“Kindes- und Erwachsenenschutzrecht” [German abbreviation KESR] also referred to as “the new law” for the involuntary commitment [as in effect since January 1^st^, 2013, based on Art. 426 ff. ZGB]; KPPP, Clinic for Psychiatry, Psychotherapy and Psychosomatics at PUK; PUK, Zurich University Hospital of Psychiatry; SAMW, Swiss Academy of Medical Sciences; Tab., table; vs., versus; ZGB, Swiss Civil Code

## References

[1] Schweizerische Akademie der Medizinischen Wissenschaften (SAMW) (2015). Zwangsmassnahmen in der Medizin. Medizinisch-ethische Richtlinien der SAMW.

[2] Schweizerisches Zivilgesetzbuch vom 10. Dezember 1907. https://www.admin.ch/opc/de/classified-compilation/19070042/201407010000/210.pdf. Accessed 10 Aug 2016.

[3] Gassmann J (2011). Wirksamkeit des Rechtsschutzes bei psychiatrischen Zwangseinweisungen in der Schweiz.

[4] Vögeli D. Schwelle für Zwangseinweisungen umstritten. Im Kanton Zürich erfolgt mindestens ein Viertel aller psychiatrischen Hospitalisierungen unfreiwillig. Neue Zürcher Zeitung (NZZ), Nr.22. 28. Januar 2013.

[5] Maier T (2001). Die Praxis der Fürsorgerischen Freiheitsentziehung. Praxis.

[6] Jaeger M, Ospelt I, Kawohl W, Theodoridou A, Roessler W, Hoff P (2014). Qualität unfreiwilliger Klinikeinweisungen in der Schweiz. Praxis.

[7] Rosch D (2011). Die fürsorgerische Unterbringung im revidierten Kindes- und Erwachsenenschutzrecht. Aktuelle juristische Praxis..

[8] Zivilgesetzbuch, Obligationenrecht, SchKG, BV und weitere Erlasse, 12. Auflage 2015/2016, Orell Füssli Verlag AG, Zürich.

[9] Geiser T, Reusser RE, (Hrsg.) (2012). Basler Kommentar Erwachsenenschutz.

[10] Swanson JW, Swartz MS, Elbogen EB, Van Dorn RA, Wagner HR, Moser LA (2008). Psychiatric advance directives and reduction of coercive crisis interventions. J Ment Health.

[11] Christen L, Christen S (2005). Beschreibung der Basisdaten stationärer psychiatrischer Kliniken der Schweiz. Analyse der Psychiatrie-Zusatzdata 2000-2002. Arbeitsdokument 13 des Obsan.

[12] Nawka A (2013). Gender differences in coerced patients with schizophrenia. BMC Psychiatry.

[13] Riecher-Roessler A, Roessler W (1993). Compulsory admission of psychiatric patients- an international comparison. Acta Psychiatr Scand.

[14] Psychiatrische Universitätsklinik Zürich. Jahresbericht 2013, Strategien. Zurich: PUK; 2014.

[15] Psychiatrische Universitätsklinik Zürich. Aktuelles: Schweizweit grösste Klinik für Erwachsenenpsychiatrie. Medienmitteilung vom 31. Januar. 2013. http://www.pukzh.ch/default/assets/File/Medienmitteilung%20Fusion%20KPPP_Schaffung%20KFP_20130201_definitiv_für%20Website%20und%20Intranet.pdf. Accessed 2 Feb 2015.

[16] Gesundheitsdirektion des Kanton Zürich: Leitfaden zum neuen Kindes- und Erwachsenenschutzrecht für Ärztinnen und Ärzte (November 2012). http://www.zh.ch/internet/gesundheitsdirektion/de/themen/berufe/aerzte.html. Accessed 15 Feb 2015.

[17] Guler A. Die wichtigsten Neuerungen des Kindes- und Erwachsenenschutzrechts, Vortrag, gehalten am 08.11.2012 in Aarau beim ZSBA. http://www.zsba.ch/ZSBA%20Daten/2012/Manuskript.pdf. Accessed 25 Jan 2015.

[18] Bundesverfassung der Schweizerischen Eidgenossenschaft vom 18. April 1999. http://www.admin.ch/opc/de/classified-compilation/19995395/201303030000/101.pdf. Accessed 25 Jan 2015.

[19] Bridler R (2013). Einen Schritt vor, zwei Schritte zurück. Das neue Erwachsenenschutzrecht und die Psychiatrie. Schweizerische Ärztezeitung.

[20] Patientinnen- und Patientengesetz Kanton Zürich vom 05. April 2004 (813.13). http://www2.zhlex.zh.ch/Appl/zhlex_r.nsf/0/0672AD30D614A770C1256ED00042BDFD/$file/813.13.pdf. Accessed 15 Feb 2015.

[21] Zürcher Gesetzessammlung: Einführungsgesetz zum Kindes- und Erwachsenenschutzrecht (EG KESR) vom 25. Juni 2012. http://www2.zhlex.zh.ch/appl/zhlex_r.nsf/0/9AF3F325F5CFFEE7C1257A930023CA6E/$file/232.3.pdf admission before the revision E7C1257A930023CA6E/$file/232.3.pdf. Accessed 26 Jan 2015.

[22] Link BG, Andrews H, Cullen FT (1992). The violent and illegal behavior of mental patients reconsidered. Am Sociol Rev.

[23] Steinert T (1998). Schizophrenia and violence: epidemiological, forensic and clinical aspects. Fortschritte der Neurologie, Psychiatrie, und ihrer Grenzgebiete.

[24] Kallert TW, Katsakou C, Adamowski T, Dembinskas A, Fiorillo A, Kjellin L (2011). Coerced hospital admission and symptom change- a prospective observational multi-centre study. PLoS One.

